# Genetically Supported Causality Between Micronutrients and Sleep Behaviors: A Two‐Sample Mendelian Randomization Study

**DOI:** 10.1002/brb3.70237

**Published:** 2025-02-05

**Authors:** Ruijie Zhang, Liyan Luo, Lu Zhang, Xinao Lin, Chuyan Wu, Feng Jiang, Jimei Wang

**Affiliations:** ^1^ Department of Neonatology Obstetrics and Gynecology Hospital of Fudan University Shanghai China; ^2^ Department of Neonatology Dali Bai Autonomous Prefecture Maternal and Child Health Care Hospital Dali China; ^3^ Department of Rehabilitation Medicine The First Affiliated Hospital of Nanjing Medical University Nanjing China

**Keywords:** chrononutrition, chronotype, Mendelian randomization, micronutrients, sleep behaviors

## Abstract

**Background:**

Sleep behaviors, defined by the total duration of sleep and chronotype, significantly influence overall health. Compromised sleep quality, which is often manifested through reduced sleep duration and the prevalence of insomnia, has been found to be associated with micronutrient deficiencies. Nonetheless, the existence of a causal relationship between micronutrient levels and sleep behaviors remains to be established.

**Methods:**

A two‐sample Mendelian randomization (MR) analysis, utilizing data from genome‐wide association studies (GWAS), was employed to examine the associations between 15 micronutrients (copper; calcium; carotene; folate; iron; magnesium; potassium; selenium; vitamins A, B12, B6, C, D, and E; and zinc) and various sleep behaviors, including short and long sleep durations, insomnia, and chronotype. Furthermore, multivariable MR (MVMR) analysis was performed to address potential confounding due to the interrelationships among micronutrients and to discern potential causal relationships.

**Results:**

The MR analysis identified a causal association between folate levels and chronotype (odds ratio [OR] = 1.09; 95% confidence interval [CI]: 1.01–1.17; *p* = 0.02), indicating a tendency toward morningness. Conversely, vitamin B6 (OR = 0.91; 95% CI: 0.86–0.96; *p* = 1.05 × 10^−3^) and vitamin D (OR = 0.94; 95% CI: 0.88–1.00; *p* = 0.03) showed inverse associations with chronotype, indicative of a preference for eveningness. MVMR analysis confirmed the positive association between folate (OR = 1.286, 95% CI = 1.124–1.472, *p* < 0.001) and chronotype and a negative association with vitamin B6 (OR = 0.750, 95% CI = 0.648–0.868, *p* < 0.001). No causal relationships were established between micronutrient levels and either sleep duration or insomnia.

**Conclusions:**

Elevated folate levels correlate with morning‐type preferences (“morning birds”), while higher concentrations of vitamin B6 are associated with evening‐type preferences (“evening owls”).

## Graphical abstract

We employed two‐sample Mendelian randomization to explore the causal relationships between sleep behaviors and micronutrition in GWAS data. We found that folate levels are associated with a morning‐oriented chronotype, while elevated levels of vitamin B6 and vitamin D are associated with an evening‐oriented chronotype.

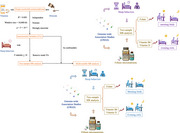

## 1 Introduction

Sleep patterns are defined by their length, often calculated as the total amount of sleep within a 24‐h cycle, and by chronotype. Chronotype refers to an individual's natural predisposition to be active at particular times during a 24‐h cycle (Günal 2023). “Morning types,” commonly known as “larks,” tend to be more energetic and productive in the early morning and typically prefer to go to bed and wake up early. “Evening types,” often referred to as “owls,” are more active and focused in the evening, usually preferring to go to bed and rise later (Günal 2023; Wu et al. 2024). Numerous factors, including age, gender, and ethnicity, influence sleep behavior (Hirshkowitz et al. 2015; Grandner 2019; Walch, Cochran, and Forger 2016). Notably, the duration and quality of sleep are significantly determined by genetic factors. Meta‐analyses have demonstrated that approximately 46% of the variance in sleep duration among individuals is attributable to genetic differences, and 44% of the variance in sleep quality is determined by genetics (Breitenstein, Doane, and Lemery‐Chalfant 2021). This finding is corroborated by genetic studies that employed genome‐wide association studies (GWAS) to analyze self‐reported sleep duration data from the UK Biobank. The results were validated for consistency in separate research involving both adults and children/adolescents (Kocevska et al. 2021; Dashti et al. 2019).

The impact of micronutrient intake on sleep has received less attention than that of macronutrients. However, emerging studies suggest that shorter sleep durations in adults are associated with deficiencies in several micronutrients, specifically calcium, magnesium, and vitamins D and K (Ikonte et al. 2019; Al Hinai et al. 2024). Furthermore, numerous clinical trials have provided evidence supporting the assertion that adequate concentrations of zinc and iron can enhance sleep quality in infants and adolescents (Innocenti et al. 2023; Ji and Liu 2015; Kordas et al. 2009). Traditional observational studies may be influenced by various confounding factors, which could introduce biases. To date, there has not been a comprehensive review of the literature examining the connection between micronutrients and sleep behaviors from a developmental standpoint.

Mendelian randomization (MR) utilizes single nucleotide polymorphism (SNP) statistics from GWAS to infer possible causal connections among various complex traits (Gupta, Walia, and Sachdeva 2017; Yao et al. 2022). SNPs, due to their random assignment during meiosis and fertilization prior to birth, are less susceptible to alterations by disease conditions, environmental factors, or other confounders (Davies, Holmes, and Davey Smith 2018). Therefore, they may offer a more precise understanding of true causal relationships compared to conventional observational studies.

In our research, we investigated the genetic underpinnings and potential causal links between micronutrients and sleep behaviors. This was achieved by examining genetic correlations and polygenic overlaps using GWAS summary statistics. Additionally, we employed two‐sample and multivariable MR (MVMR) analyses to explore the possible causal effects of micronutrients on sleep behaviors.

## 2 Methods

### 2.1 Study Design

**FIGURE 1:**
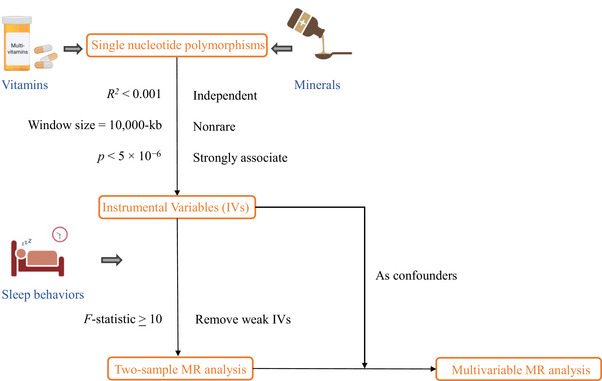
A flow chart of the study design.

A schematic overview of the research design is depicted in Figure 1. We conducted an MR investigation utilizing data from 18 publicly accessible GWAS to acquire summary statistics. In the two‐sample MR analysis, data concerning sleep behaviors were sourced from three independent GWAS consortia. These data were employed for initial analyses and replication studies, and ultimately integrated into a meta‐analysis to consolidate findings. For the MVMR analysis, we investigated the causal relationships between various micronutrients and sleep behaviors. To increase the statistical robustness of our findings, we combined estimates from various data sources. All studies involved in the GWAS had previously been approved by the appropriate review boards, and no further ethical approval or participant consent was necessary for this analysis.

### 2.2 GWAS Data of Sleep‐Related Behaviors

Participants recorded their own sleep duration by noting the total hours slept every 24 h, including naps, measured in hourly increments. We carried out distinct GWAS for individuals of European descent, categorizing them based on their sleep duration. This included a group with short sleep duration (less than 7 h per night, *n* = 106,192 cases) and another with long sleep duration (more than 9 h per night, *n* = 34,184 cases). These were compared to a control group that reported sleeping between 7 and 9 h per night (*n* = 305,742) (Dashti et al. 2019). We excluded responses that reported extremely low (less than 3 h) or extremely high (more than 18 h) sleep durations. Responses marked as “Do not know” or “Prefer not to answer” were considered missing data. We can obtain the GWAS summary statistics from the Sleep Disorder Knowledge Portal at https://sleep.hugeamp.org/downloads.html.

To assess chronotype, subjects responded to the question, “Do you consider yourself to be?” with choices spanning from “Definitely a ‘morning’ person” to “Definitely an ‘evening’ person.” Responses were scored, with “Definitely a ‘morning’ person” and “More a ‘morning’ than ‘evening’ person” classified as cases, assigned two and one points, respectively. Conversely, those identifying as “Definitely an ‘evening’ person” or “More an ‘evening’ than ‘morning’ person” were designated as controls, with scoring of −2 and −1 points, respectively. The study by Jones et al. (2019) encompassed 252,287 cases and 150,908 controls of European ancestry. The GWAS summary statistics for this analysis are also available on the Sleep Disorder Knowledge Portal.

In the assessment of insomnia, participants answered the question: “Do you have trouble falling asleep at night or do you wake up in the middle of the night?” The response options provided were “never/rarely,” “sometimes,” “usually,” or “prefer not to answer.” Participants who chose “usually” were identified as cases of insomnia, while those who answered “never/rarely” were designated as controls. The GWAS, carried out by Watanabe et al. (2022), involved 593,724 cases and 1,771,286 controls of European descent and identified 554 loci associated with insomnia. The GWAS summary statistics are available from https://ctg.cncr.nl/software/summary_statistics/.

In our research, we conducted a comprehensive search through PubMed and the IEU OpenGWAS project (https://gwas.mrcieu.ac.uk/) to identify the most recent large‐scale GWAS studies on micronutrients, with the final search conducted in April 2024. To avoid overlap between the exposures and outcomes in our study, we excluded certain micronutrients that were obtained from these specific databases during our data collection. We observed a notable absence of genomic studies concerning chloride; fluoride; phosphorus; sulfur; vitamins B1, B2, B3, B5, and B7; and iodine. Furthermore, studies on vitamin K, cobalt, chromium, sodium, and molybdenum were not included due to their inconclusive genomic results (Dashti et al. 2014; Ng et al. 2015). This selection process led us to preliminarily identify 15 micronutrients for further investigation: copper (Evans et al. 2013), calcium (O'Seaghdha et al. 2013), carotene (Ferrucci et al. 2009), folate (Grarup et al. 2013), iron (Bell et al. 2021), magnesium (Meyer et al. 2010), potassium (Meyer et al. 2010), selenium (Evans et al. 2013), vitamin A (Mondul et al. 2011), vitamin B12 (Grarup et al. 2013), vitamin B6 (Hazra et al. 2009), vitamin C (Zheng et al. 2020), vitamin D (Revez et al. 2020), vitamin E (Major et al. 2011), and zinc (Evans et al. 2013).

### 2.3 Two‐Sample MR Analysis

In our research, we utilized the two‐sample MR approach, employing the Two Sample MR (version 0.5.11) package (Hemani, Tilling, and Smith 2017; Hemani et al. 2018). Our principal technique for MR analysis was the inverse variance weighting (IVW) method. This meta‐analytic approach combines Wald estimates from each SNP to compute overall effect estimates of the exposure on the outcome (Burgess, Butterworth, and Thompson 2013). This method operates under the assumption that all included variants are valid instruments, or that any horizontal pleiotropy is balanced, thus not violating the fundamental assumptions of MR.

We supplemented our primary analysis with several auxiliary methods to further assess causality. The MR‐Egger method (Bowden, Davey Smith, and Burgess 2015) uses weighted linear regression to address small study biases by analyzing the slope of outcome coefficients against exposure coefficients. It offers reliable causal estimates even when all genetic variants may be invalid instruments, albeit with less efficiency compared to IVW and median‐based methods. The weighted median approach integrates data from multiple genetic variants to produce a single causal estimate and tolerates up to 50% of the data originating from potentially invalid instruments. SNPs were classified with mode‐based methods (Hartwig, Davey Smith, and Bowden 2017) into clusters by similarity in causal effects, estimating the causal effect based on the most populous clusters. This approach minimizes bias and reduces type‐I errors, particularly when the SNPs in the largest cluster are valid instruments.

To improve the trustworthiness of our findings, we undertook an extensive series of sensitivity checks. First, we evaluated the heterogeneity of SNP effects, which could bias the IVW estimates, by computing Cochran's Q statistics (Bowden et al. 2019). A *p*‐value above 0.05 for this test suggested minimal heterogeneity impact. Where heterogeneity was detected, we employed the random effects model of IVW to assess the causal effects more appropriately. Second, to address the risk of horizontal pleiotropy, which could violate the exclusion restriction assumption of Mendel's Third Law instrumental variables (IVs) should not influence the outcome except through the exposure (Didelez and Sheehan 2007; Angrist, Imbens, and Rubin 1996), we analyzed the MR‐Egger regression intercept. An intercept not significantly differing from zero (*p* > 0.05) indicated an absence of horizontal pleiotropy (Burgess and Thompson 2017). Third, the MR Steiger directional test was utilized to verify the assumption that the exposure influences the outcome, rather than the reverse (Hemani, Tilling, and Smith 2017). This test compares the *R* ^2^ values for the outcome and exposure explained by the IVs. A significantly lower *R* ^2^ for the outcome than the exposure (MR Steiger test *p* < 0.05) suggested the absence of reverse causality between the two. Lastly, we implemented a leave‐one‐out analysis to ascertain the robustness of the causal effect. This approach involves sequentially removing each IV and recalculating the IVW using the remaining IVs, thus assessing the influence of individual variants on the overall causal estimate. These methods collectively ensure our analysis is both thorough and reliable in detecting true causal relationships.

### 2.4 Multivariable MR Analysis

In this study, we acknowledged the potential for confounding among various vitamins and minerals when using two‐sample or standard MR due to the pleiotropic effects that one micronutrient may exert on others. To address this issue, we employed MVMR analysis. This approach integrates a pleiotropy correction by including all pertinent sleep behaviors in one model, which helps reduce bias (Sanderson 2021). For the MVMR analysis, we utilized all the variants from the two‐sample MR of micronutrients as IVs. This analysis was conducted using the “Mendelian Randomization” R package (version 0.10.0). Within this framework, both the IVW and MR‐Egger methods were employed to determine the causal relationships between one sleep behavior and the other two.

To further assess heterogeneity in the outcomes of SNP, we calculated an adjusted Cochran's Q statistic using summary data (Sanderson et al. 2019), which helps identify variations in SNP effects that could impact the validity of our MR estimates. Additionally, the presence of pleiotropic effects was assessed by evaluating the MR‐Egger intercept term to determine whether the IVs had any effects on the outcome that were not mediated through the exposure. As our primary method, IVW was used under the assumption of no pleiotropic effects. However, when directional pleiotropy was suspected, we employed the multivariable MR‐Egger method, which is specifically designed to accommodate such pleiotropy (Rees, Wood, and Burgess 2017). This comprehensive approach allows us to more accurately discern the direct and indirect effects of sleep behaviors on lifespan within a multivariable framework.

## 3 Results

### 3.1 Two‐Sample MR Analysis

**FIGURE 2:**
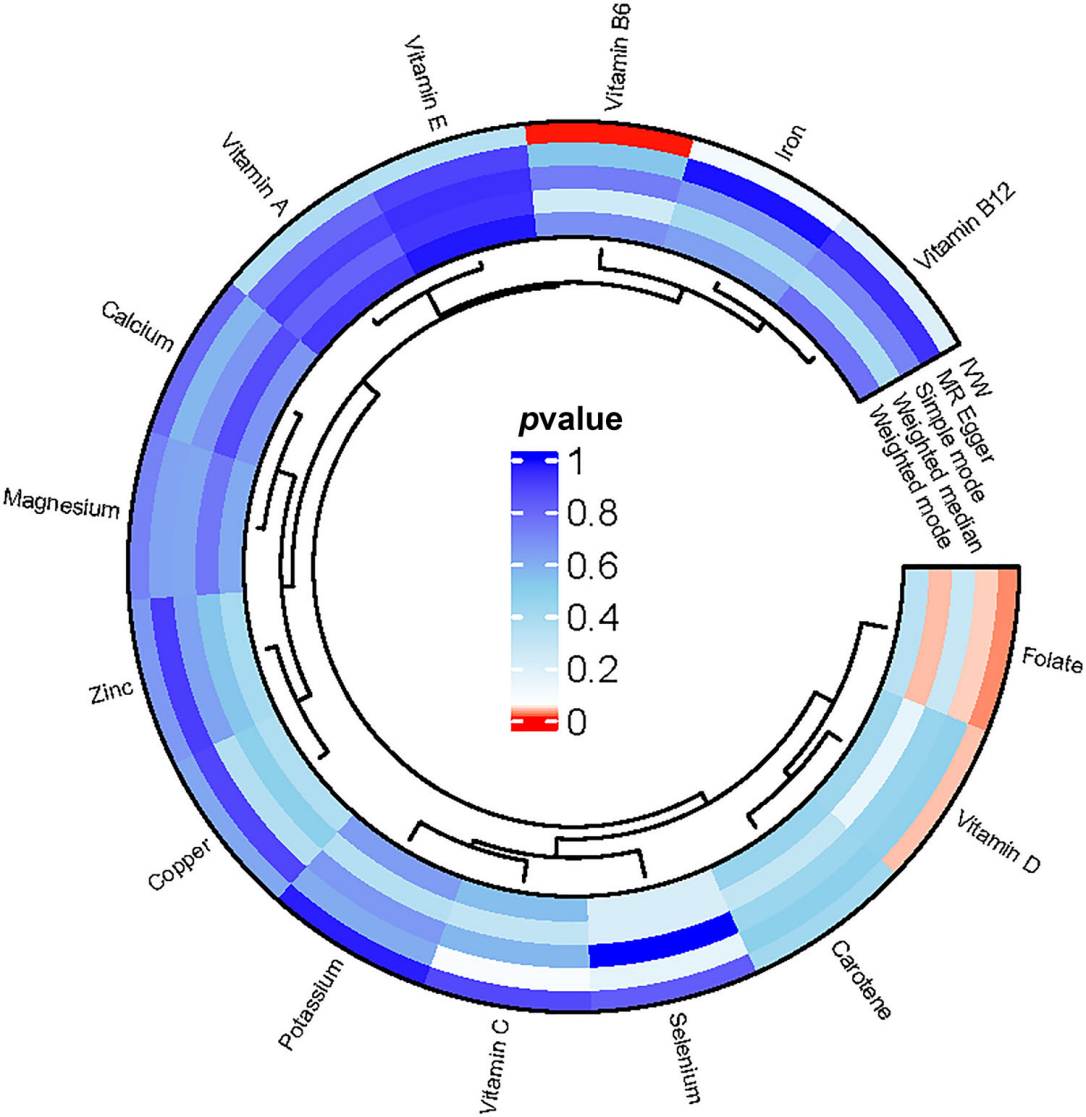
Circos image of the associations of the 15 micronutrients with the chronotype from five statistic method. Red indicates a *p* value of less than 0.05.

To explore the connections between micronutrients and various sleep behaviors, we utilized a two‐sample MR approach. We identified significant associations with chronotype for folate and vitamins B6 and D (*p* < 0.05) (Table S1; Figure 2). However, no significant correlations were observed between chronotype and the levels of calcium; carotene; copper; iron; magnesium; phosphorus; potassium; selenium; vitamins A, B12, C, and E; and zinc in the bloodstream (Table S1; Figure 2). Additionally, the MR analysis did not establish causal relationships between circulating micronutrients and the three sleep behaviors of short sleep duration, long sleep duration, and insomnia (Tables S2–S4).

**FIGURE 3:**
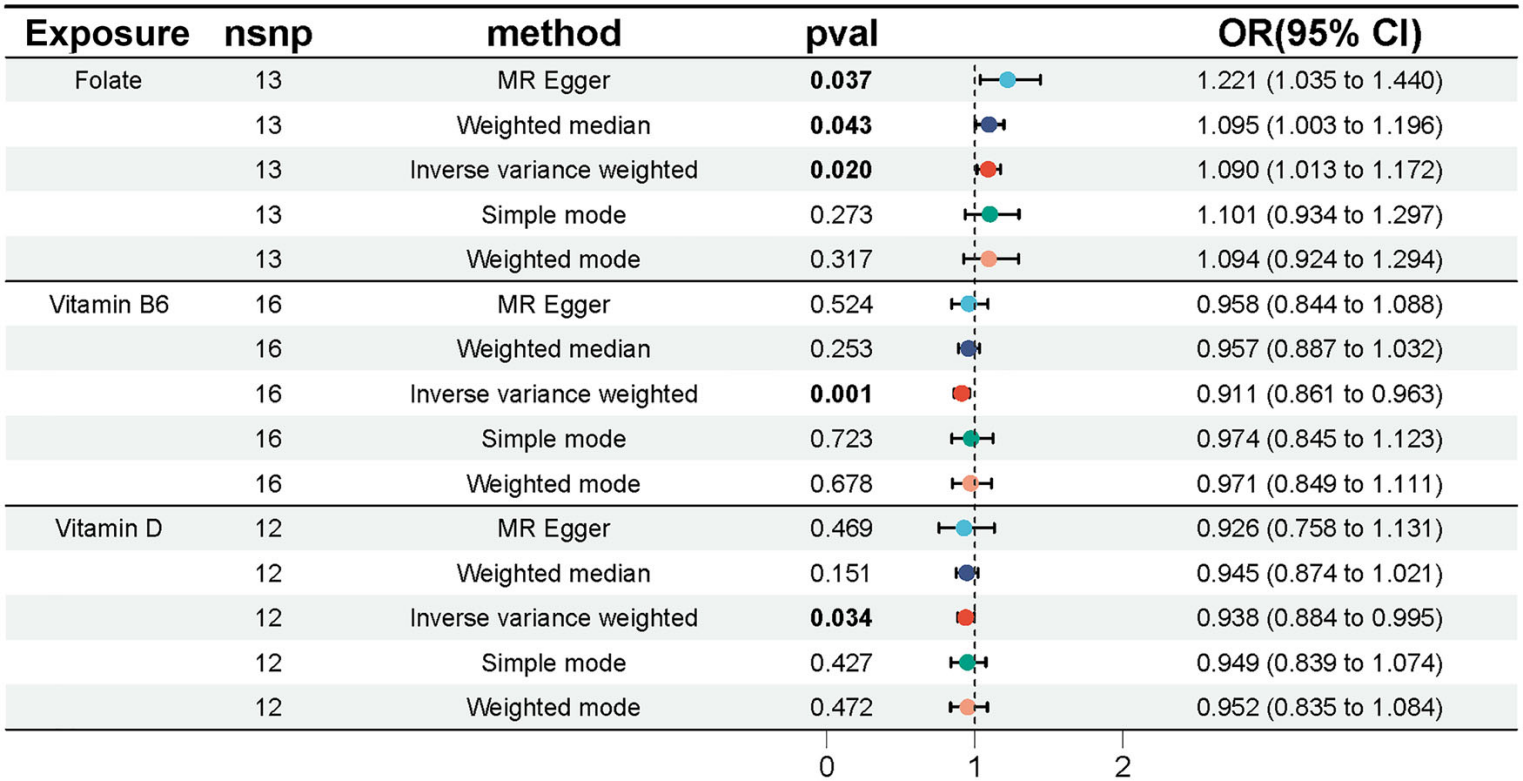
Two‐sample MR analysis on the association between folate, vitamin B6, and vitamin D levels and chronotype. Abbreviations: CI, confidence interval; IVW, inverse‐variance weighted; nsnp, number of SNP; OR, odds ratio.

Specifically, for each standard deviation (SD) increase in folate levels, the odds ratio (OR) is 1.09, with a 95% confidence interval (CI) ranging from 1.01 to 1.17 (*p* = 0.02). The MR‐Egger and weighted median approaches yielded congruent results, reinforcing the relationship. Specifically, the MR‐Egger method reported an OR of 1.22 with a 95% CI ranging from 1.03 to 1.44 and a *p*‐value of 0.04. The weighted median analysis showed an OR of 1.10 with a 95% CI of 1.10 to 1.19 and a *p*‐value of 0.03, as detailed in Table S1. Additionally, vitamins B6 and D were negatively correlated with chronotype. For each standard deviation increase, the ORs were 0.91 with a 95% CI of 0.86 to 0.96 (*p* = 0.001) and 0.94 with a 95% CI of 0.88 to 1.00 (*p* = 0.03), respectively (Figures 2 and 3).

**FIGURE 4:**
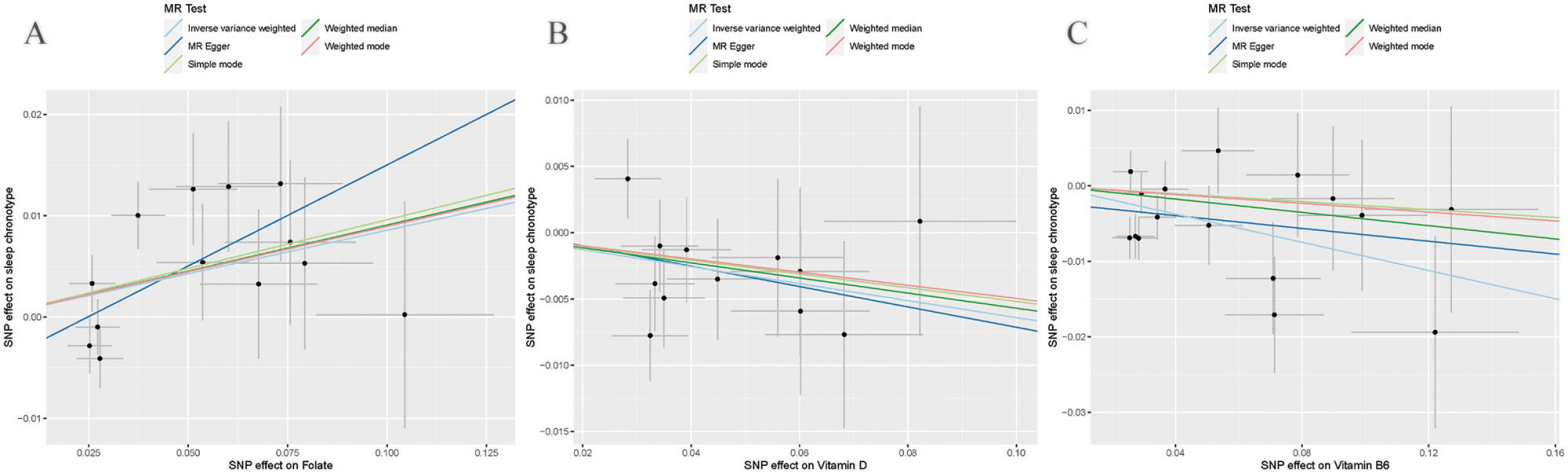
Scatter plot illustrating the two‐sample MR results for the effects of folate (A), vitamin B6 (B), and vitamin D (C) on chronotype. The slope of various colorful lines illustrates the estimated MR effect derived from different MR methods.

**FIGURE 5:**
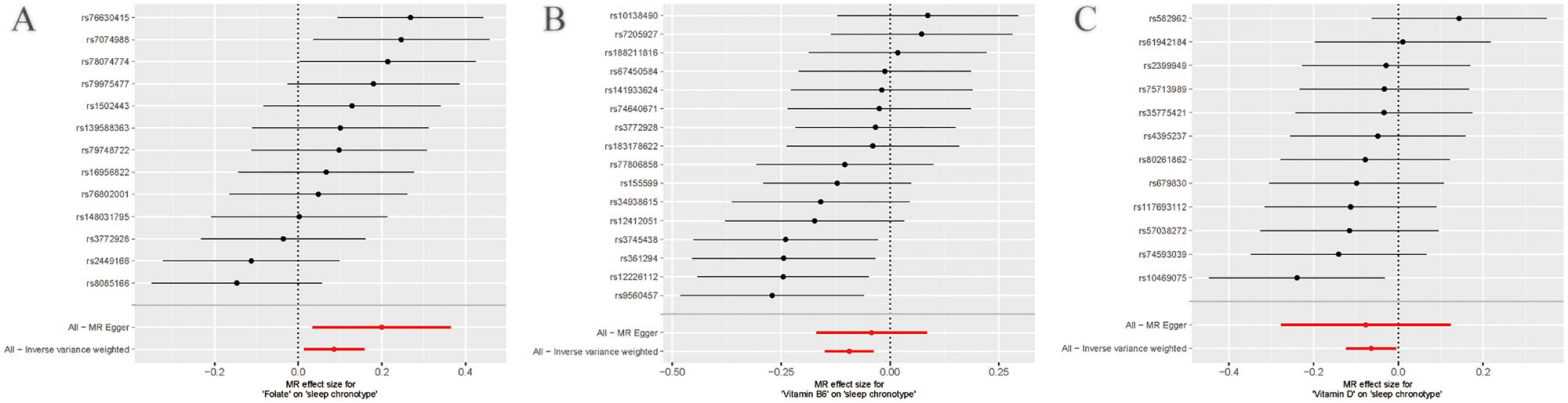
Forest plot of folate (A), vitamin B6 (B), and vitamin D (C) on chronotype. Each line represents the effect of an IV.

**FIGURE 6:**
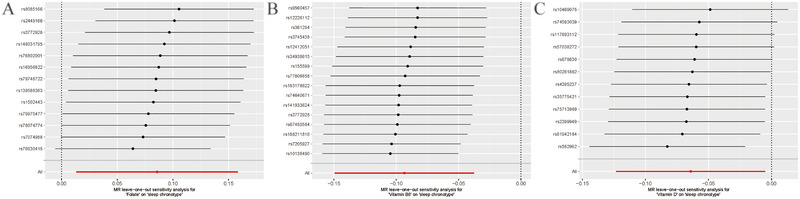
Forest plot of leave‐one‐out result of folate (A), vitamin B6 (B), and vitamin D (C). Each line represents the IVW estimate of the impact of short sleep duration on lifespan after excluding this specific SNP. The absence of any line crossing zero suggests that the result is robust.

MR results are presented in scatter plots with different color‐coded trend lines to showcase estimates calculated from the various methods, as seen in Figure 4. Additionally, forest plots depict the influence of each IV of folate, vitamin B6, and vitamin D levels on chronotype (Figure 5). To mitigate potential biases related to pleiotropy and the impact of individual IVs, additional analyses were performed. The Cochran's Q statistic revealed no significant heterogeneity among the IVs, with results for folate showing *Q* = 19.54, differences (df) = 21, *p* = 0.08; for vitamin B6, *Q* = 18.65, df = 15, *p* = 0.23; and for vitamin D, *Q* = 8.54, df = 11, *p* = 0.66. Furthermore, no significant evidence of horizontal pleiotropy was found by the MR‐Egger regression, as demonstrated in Table S2. The robustness of these findings is further supported by the leave‐one‐out analysis, as shown in Figure 6, confirming that no single IV disproportionately influenced the overall causal assessment.

### 3.2 Multivariable MR

**FIGURE 7:**
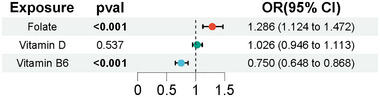
MVMR results for sleep behaviors, conditioned on chronotype.

Given the interrelationships among folate, vitamin B6, and vitamin D, the two‐sample MR analysis could not exclude potential confounding because of the other micronutrients. Thus, we conducted an MVMR analysis, estimating the direct effect of each exposure conditional on the others included in the model (Sanderson 2021). In this more comprehensive analysis, folate showed a positive causal effect on chronotype (OR = 1.286, 95% CI = 1.124–1.472, *p* < 0.001), while vitamin B6 demonstrated a negative causal effect (OR = 0.750, 95% CI = 0.648–0.868, *p* < 0.001). The impact of vitamin D on chronotype remains unclear (Table S5; Figure 7).

## 4 Discussion

Through our two‐sample MR analyses, we identified associations between specific circulating micronutrients—folate, vitamin B6, and vitamin D—and chronotype. Folate exhibited a positive correlation with chronotype, suggesting that adults possessing elevated levels of folate tend to be morning‐oriented individuals (“early birds”). Conversely, higher concentrations of vitamins B6 and D were associated with a greater likelihood of being evening‐oriented (“night owls”). However, no causal relationships were established between these micronutrients and sleep disorders such as short sleep duration, long sleep duration, or insomnia.

Our MVMR analysis found a positive correlation between folate levels and chronotype, whereas vitamin B6 was negatively correlated with chronotype. The impact of vitamin D on chronotype remains unclear. Further analysis confirmed that individuals with higher levels of folate are more likely to exhibit an early‐to‐bed, early‐to‐rise pattern, while those with elevated levels of vitamin B6 tend to have a late‐to‐bed, late‐to‐rise sleep pattern.

Sleep habits are interconnected with the composition and timing of food intake. Morning chronotypes tend to have higher intakes of energy, protein, and fats, with lower carbohydrate consumption (Günal 2023). Posterior results show that a healthier dietary structure, characterized by closer alignment with the Mediterranean diet and reduced consumption of sugary drinks and juices, correlates with a decreased incidence of poor sleep (Huang et al. 2024). Although meta‐analyses suggest that no specific foods explicitly impact sleep, the timing of food intake does affect the chronotype (Netzer, Strohl, and Pramsohler 2024). The results demonstrate that eating dinner after 10 p.m. leads to diminished lipase activity. Furthermore, the sleep–wake cycle influences the effect of lipolytic enzymes on metabolism (Arredondo‐Amador et al. 2020).

Extensive research highlights the protective effects of vitamin D on sleep quality, though its influence on chronotype is not well‐defined (Ji, Grandner, and Liu 2017). For instance, a study of postmenopausal women indicated that those with higher dietary intake of vitamin D experienced a delay in sleep phase onset (Grandner et al. 2010). Conversely, research in Japan involving young women aged 18–20 years revealed a negative correlation between the midpoint of sleep and the intake of several nutrients, including potassium, calcium, magnesium, iron, zinc, thiamine, riboflavin, and vitamins A, D, and B (Sato‐Mito et al. 2011). The findings suggest that higher physiological levels of folate and vitamins D and B6 are linked to earlier sleep initiation and wake times (Jones et al. 2019).

Consistent with other studies, our research did not identify a significant association between sleep duration and the levels of folate, vitamin B6, or zinc (Ikonte et al. 2019). Nevertheless, these micronutrients, which are known to enhance melatonin synthesis, contribute to regulating sleep and circadian rhythms (Peuhkuri, Sihvola, and Korpela 2012). Iron and zinc are particularly crucial in enhancing the sleep quality of children and adolescents, which can significantly impact cognitive development due to issues related to sleep quality (Ji et al. 2021; Liu et al. 2024). A study conducted in Turkey revealed that individuals with an evening‐type (E‐type) chronotype consume more vitamin B6 compared to those with a morning‐type (M‐type) chronotype (Toktaş, Erman, and Mert 2018). This increased intake may lead to a compensatory accumulation of vitamin B6 in the body, resulting in higher concentrations in the bloodstream.

It is crucial to highlight some limitations of our study. In the first place, our findings, derived from a population of European descent, may not be generalizable to other racial groups. Second, the classification of short sleep duration in our study was based on self‐reports rather than objective measurements, which could potentially introduce bias into the GWAS results. With the increasing prevalence of smart wearable devices, future research could potentially achieve more precise measurements of sleep conditions.

## 5 Conclusion

Our two‐sample Mendelian randomization analysis suggests that individuals with higher folate levels tend to be morning‐oriented, which may be beneficial for enhancing sleep quality. Conversely, those with elevated levels of vitamin B6 and vitamin D tend to be evening‐oriented. Future research should include diverse populations and also consider conducting controlled, randomized trials to precisely determine micronutrient intake levels, providing a robust framework to validate the conclusions derived from Mendelian randomization.

## Author Contributions

**Ruijie Zhang**: writing–original draft, methodology, investigation. **Liyan Luo**: supervision. **Lu Zhang**: writing–review and editing, methodology, investigation. **Xinao Lin**: writing–review and editing, software. **Chuyan Wu**: supervision. **Feng Jiang**: conceptualization, methodology, writing–review and editing. **Jimei Wang**: funding acquisition, supervision, project administration.

## Ethics Statement

All the GWAS data utilized in this research were sourced from publicly accessible databases, and no original data were collected for this study. Each of the studies included had received approval from their respective institutional ethics review committees. Additionally, informed consents, both for participation and publication, were obtained from all participants involved.

## Consent

All authors consent to publication.

## Conflicts of Interest

The authors declare no conflicts of interest.

### Peer Review

The peer review history for this article is available at https://publons.com/publon/10.1002/brb3.70237.

## Supporting information



Supplementary Materials.

Supplementary Materials.

Supplementary Materials.

Supplementary Materials.

Supplementary Materials.

## Data Availability

The datasets used and/or analyzed during the current study are available from the corresponding author upon reasonable request.
