# Supplementary material for: Genetically Supported Causality Between Micronutrients and Sleep Behaviors: A Two‐Sample Mendelian Randomization Study
Source: Brain Behav. 2025 Feb 5;15(2):e70237. doi: 10.1002/brb3.70237 (PMC11799067; doi:10.1002/brb3.70237)
Supplement: Supplementary file 1 — Supplementary Materials. [file BRB3-15-e70237-s005.docx]

Table S1. Two sample MR analysis of the association between circulating micronutrients and sleep chronotype.

| **Exposure** | **Method** | **No. of SNPs** | ***P* value** | ***OR* (95% *CI*)** |
| --- | --- | --- | --- | --- |
| Calcium | IVW | 20 | 0.79 | 0.99 (0.93, 1.06) |
|  | MR Egger | 20 | 0.57 | 1.06 (0.87, 1.31) |
|  | Simple mode | 20 | 0.67 | 1.03 (0.90, 1.19) |
|  | Weighted median | 20 | 0.87 | 1.01 (0.94, 1.08) |
|  | Weighted mode | 20 | 0.67 | 1.03 (0.90, 1.18) |
| Carotene | IVW | 15 | 0.41 | 0.98 (0.93, 1.03) |
|  | MR Egger | 15 | 0.48 | 1.04 (0.93, 1.16) |
|  | Simple mode | 15 | 0.43 | 0.95 (0.85, 1.07) |
|  | Weighted median | 15 | 0.29 | 0.96 (0.89, 1.03) |
|  | Weighted mode | 15 | 0.44 | 0.95 (0.85, 1.07) |
| Copper | IVW | 6 | 0.61 | 1.00 (0.99, 1.02) |
|  | MR Egger | 6 | 0.89 | 1.00 (0.98, 1.03) |
|  | Simple mode | 6 | 0.35 | 1.01 (0.99, 1.04) |
|  | Weighted median | 6 | 0.48 | 1.01 (0.99, 1.02) |
|  | Weighted mode | 6 | 0.36 | 1.01 (0.99, 1.03) |
| Folate | IVW | 13 | 0.02 | 1.09 (1.01, 1.17) |
|  | MR Egger | 13 | 0.04 | 1.22 (1.03, 1.44) |
|  | Simple mode | 13 | 0.27 | 1.10 (0.93, 1.30) |
|  | Weighted median | 13 | 0.03 | 1.10 (1.01, 1.19) |
|  | Weighted mode | 13 | 0.32 | 1.09 (0.92, 1.30) |
| Iron | IVW | 12 | 0.10 | 0.94 (0.88, 1.01) |
|  | MR Egger | 12 | 0.99 | 1.00 (0.80, 1.26) |
|  | Simple mode | 12 | 0.66 | 0.97 (0.84, 1.12) |
|  | Weighted median | 12 | 0.4 | 0.96 (0.89, 1.05) |
|  | Weighted mode | 12 | 0.63 | 0.97 (0.85, 1.10) |
| Magnesium | IVW | 17 | 0.72 | 0.99 (0.94, 1.04) |
|  | MR Egger | 17 | 0.63 | 0.98 (0.88, 1.08) |
|  | Simple mode | 17 | 0.61 | 1.03 (0.91, 1.17) |
|  | Weighted median | 17 | 0.75 | 1.01 (0.94, 1.08) |
|  | Weighted mode | 17 | 0.61 | 1.03 (0.91, 1.17) |
| Potassium | IVW | 14 | 0.97 | 1.00 (0.92, 1.08) |
|  | MR Egger | 14 | 0.60 | 0.95 (0.77, 1.16) |
|  | Simple mode | 14 | 0.65 | 0.96 (0.83, 1.13) |
|  | Weighted median | 14 | 0.34 | 0.96 (0.89, 1.04) |
|  | Weighted mode | 14 | 0.64 | 0.96 (0.83, 1.12) |
| Selenium | IVW | 6 | 0.83 | 1.00 (0.98, 1.01) |
|  | MR Egger | 6 | 0.15 | 0.97 (0.95, 1.00) |
|  | Simple mode | 6 | 1.00 | 1.00 (0.98, 1.02) |
|  | Weighted median | 6 | 0.21 | 0.99 (0.98, 1.01) |
|  | Weighted mode | 6 | 0.21 | 0.99 (0.97, 1.00) |
| Vitamin A | IVW | 12 | 0.36 | 0.59 (0.20, 1.80) |
|  | MR Egger | 12 | 0.79 | 1.79 (0.03, 123.23) |
|  | Simple mode | 12 | 0.90 | 0.83 (0.05, 13.35) |
|  | Weighted median | 12 | 0.81 | 0.83 (0.19, 3.71) |
|  | Weighted mode | 12 | 0.91 | 0.85 (0.05, 14.23) |
| Vitamin B12 | IVW | 9 | 0.20 | 1.05 (0.98, 1.12) |
|  | MR Egger | 9 | 0.93 | 0.99 (0.83, 1.18) |
|  | Simple mode | 9 | 0.72 | 1.03 (0.88, 1.20) |
|  | Weighted median | 9 | 0.38 | 1.04 (0.95, 1.14) |
|  | Weighted mode | 9 | 0.77 | 1.02 (0.88, 1.19) |
| Vitamin B6 | IVW | 16 | 0.00 | 0.91 (0.86, 0.96) |
|  | MR Egger | 16 | 0.52 | 0.96 (0.84, 1.09) |
|  | Simple mode | 16 | 0.73 | 0.97 (0.84, 1.13) |
|  | Weighted median | 16 | 0.24 | 0.96 (0.89, 1.03) |
|  | Weighted mode | 16 | 0.68 | 0.97 (0.85, 1.11) |
| Vitamin C | IVW | 10 | 0.88 | 0.99 (0.90, 1.09) |
|  | MR Egger | 10 | 0.07 | 0.80 (0.65, 0.99) |
|  | Simple mode | 10 | 0.57 | 0.96 (0.84, 1.10) |
|  | Weighted median | 10 | 0.3 | 0.96 (0.88, 1.04) |
|  | Weighted mode | 10 | 0.54 | 0.96 (0.84, 1.09) |
| Vitamin D | IVW | 12 | 0.03 | 0.94 (0.88, 1.00) |
|  | MR Egger | 12 | 0.47 | 0.93 (0.76, 1.13) |
|  | Simple mode | 12 | 0.44 | 0.95 (0.84, 1.08) |
|  | Weighted median | 12 | 0.15 | 0.94 (0.87, 1.02) |
|  | Weighted mode | 12 | 0.42 | 0.95 (0.85, 1.07) |
| Vitamin E | IVW | 11 | 0.34 | 1.03 (0.97, 1.09) |
|  | MR Egger | 11 | 0.89 | 0.99 (0.85, 1.15) |
|  | Simple mode | 11 | 0.94 | 0.99 (0.87, 1.14) |
|  | Weighted median | 11 | 0.92 | 1.00 (0.92, 1.09) |
|  | Weighted mode | 11 | 0.98 | 1.00 (0.88, 1.14) |
| Zinc | IVW | 8 | 0.66 | 1.00 (0.99, 1.02) |
|  | MR Egger | 8 | 0.91 | 1.00 (0.94, 1.05) |
|  | Simple mode | 8 | 0.63 | 0.99 (0.96, 1.02) |
|  | Weighted median | 8 | 0.53 | 0.99 (0.98, 1.01) |
|  | Weighted mode | 8 | 0.39 | 0.99 (0.97, 1.01) |

Abbreviation: No. of SNPs, number of single nucleotide polymorphisms; OR, odds ratio; CI: Confidence Interval; IVW, Inverse variance weighted.
