# Supplementary material for: Genetically Supported Causality Between Micronutrients and Sleep Behaviors: A Two‐Sample Mendelian Randomization Study
Source: Brain Behav. 2025 Feb 5;15(2):e70237. doi: 10.1002/brb3.70237 (PMC11799067; doi:10.1002/brb3.70237)
Supplement: Supplementary file 3 — Supplementary Materials. [file BRB3-15-e70237-s004.docx]

Table S3. Two sample MR analysis of the association between circulating micronutrients and long sleep duration

| **Exposure** | **Method** | **No. of SNPs** | ***P* value** | ***OR* (95% *CI*)** |
| --- | --- | --- | --- | --- |
| Calcium | IVW | 20 | 0.10 | 1.01 (1.00, 1.03) |
|  | MR Egger | 20 | 0.39 | 1.02 (0.97, 1.07) |
|  | Simple mode | 20 | 0.63 | 0.99 (0.96, 1.02) |
|  | Weighted median | 20 | 0.89 | 1.00 (0.98, 1.02) |
|  | Weighted mode | 20 | 0.59 | 0.99 (0.97, 1.02) |
| Carotene | IVW | 15 | 0.80 | 1.00 (0.98, 1.01) |
|  | MR Egger | 15 | 0.86 | 1.00 (0.97, 1.03) |
|  | Simple mode | 15 | 0.59 | 0.99 (0.96, 1.03) |
|  | Weighted median | 15 | 0.71 | 1.00 (0.98, 1.02) |
|  | Weighted mode | 15 | 0.58 | 0.99 (0.96, 1.02) |
| Copper | IVW | 6 | 0.93 | 1.00 (1.00, 1.00) |
|  | MR Egger | 6 | 0.81 | 1.00 (0.99, 1.01) |
|  | Simple mode | 6 | 0.75 | 1.00 (0.99, 1.00) |
|  | Weighted median | 6 | 0.87 | 1.00 (1.00, 1.00) |
|  | Weighted mode | 6 | 0.66 | 1.00 (0.99, 1.00) |
| Folate | IVW | 13 | 0.92 | 1.00 (0.98, 1.01) |
|  | MR Egger | 13 | 0.95 | 1.00 (0.96, 1.04) |
|  | Simple mode | 13 | 0.61 | 1.01 (0.98, 1.04) |
|  | Weighted median | 13 | 0.53 | 1.01 (0.99, 1.03) |
|  | Weighted mode | 13 | 0.65 | 1.01 (0.97, 1.04) |
| Iron | IVW | 12 | 0.36 | 0.99 (0.97, 1.01) |
|  | MR Egger | 12 | 0.23 | 0.96 (0.91, 1.02) |
|  | Simple mode | 12 | 0.56 | 1.01 (0.97, 1.06) |
|  | Weighted median | 12 | 0.69 | 1.00 (0.98, 1.03) |
|  | Weighted mode | 12 | 0.53 | 1.01 (0.97, 1.05) |
| Magnesium | IVW | 17 | 0.95 | 1.00 (0.98, 1.01) |
|  | MR Egger | 17 | 0.23 | 1.02 (0.99, 1.05) |
|  | Simple mode | 17 | 0.77 | 1.01 (0.97, 1.04) |
|  | Weighted median | 17 | 0.76 | 1.00 (0.98, 1.02) |
|  | Weighted mode | 17 | 0.75 | 1.01 (0.97, 1.04) |
| Potassium | IVW | 14 | 0.44 | 0.99 (0.97, 1.01) |
|  | MR Egger | 14 | 0.48 | 0.98 (0.93, 1.04) |
|  | Simple mode | 14 | 0.13 | 0.96 (0.91, 1.01) |
|  | Weighted median | 14 | 0.56 | 0.99 (0.97, 1.02) |
|  | Weighted mode | 14 | 0.17 | 0.96 (0.91, 1.01) |
| Selenium | IVW | 6 | 0.87 | 1.00 (1.00, 1.00) |
|  | MR Egger | 6 | 0.63 | 1 (0.99, 1.01) |
|  | Simple mode | 6 | 0.82 | 1.00 (1.00, 1.01) |
|  | Weighted median | 6 | 0.74 | 1.00 (1.00, 1.00) |
|  | Weighted mode | 6 | 0.75 | 1.00 (1.00, 1.00) |
| Vitamin A | IVW | 12 | 0.77 | 0.96 (0.71, 1.29) |
|  | MR Egger | 12 | 0.63 | 1.34 (0.43, 4.2) |
|  | Simple mode | 12 | 0.84 | 0.93 (0.45, 1.91) |
|  | Weighted median | 12 | 0.69 | 0.92 (0.62, 1.37) |
|  | Weighted mode | 12 | 0.83 | 0.92 (0.47, 1.84) |
| Vitamin B12 | IVW | 9 | 0.64 | 1.00 (0.98, 1.01) |
|  | MR Egger | 9 | 0.41 | 0.98 (0.93, 1.03) |
|  | Simple mode | 9 | 0.87 | 1.00 (0.96, 1.04) |
|  | Weighted median | 9 | 0.87 | 1.00 (0.98, 1.03) |
|  | Weighted mode | 9 | 0.92 | 1.00 (0.96, 1.04) |
| Vitamin B6 | IVW | 17 | 0.69 | 1.00 (0.99, 1.02) |
|  | MR Egger | 17 | 0.71 | 1.01 (0.97, 1.04) |
|  | Simple mode | 17 | 0.52 | 1.01 (0.98, 1.04) |
|  | Weighted median | 17 | 0.40 | 1.01 (0.99, 1.03) |
|  | Weighted mode | 17 | 0.52 | 1.01 (0.98, 1.04) |
| Vitamin C | IVW | 10 | 0.86 | 1.00 (0.98, 1.02) |
|  | MR Egger | 10 | 0.93 | 1.00 (0.94, 1.06) |
|  | Simple mode | 10 | 0.38 | 0.98 (0.93, 1.03) |
|  | Weighted median | 10 | 0.43 | 0.99 (0.96, 1.02) |
|  | Weighted mode | 10 | 0.33 | 0.98 (0.93, 1.02) |
| Vitamin D | IVW | 13 | 0.38 | 0.99 (0.97, 1.01) |
|  | MR Egger | 13 | 0.12 | 1.05 (0.99, 1.11) |
|  | Simple mode | 13 | 0.44 | 1.02 (0.97, 1.07) |
|  | Weighted median | 13 | 0.70 | 1.00 (0.97, 1.02) |
|  | Weighted mode | 13 | 0.41 | 1.02 (0.98, 1.06) |
| Vitamin E | IVW | 12 | 0.34 | 0.99 (0.98, 1.01) |
|  | MR Egger | 12 | 0.89 | 1.00 (0.96, 1.05) |
|  | Simple mode | 12 | 0.70 | 1.01 (0.97, 1.04) |
|  | Weighted median | 12 | 0.72 | 1.00 (0.98, 1.02) |
|  | Weighted mode | 12 | 0.67 | 1.01 (0.97, 1.04) |
| Zinc | IVW | 8 | 0.14 | 1.00 (1.00, 1.01) |
|  | MR Egger | 8 | 0.43 | 1.00 (0.99, 1.02) |
|  | Simple mode | 8 | 0.37 | 1.00 (1.00, 1.01) |
|  | Weighted median | 8 | 0.16 | 1.00 (1.00, 1.01) |
|  | Weighted mode | 8 | 0.29 | 1.00 (1.00, 1.01) |

Abbreviation: No. of SNPs, number of single nucleotide polymorphisms; OR, odds ratio; CI: Confidence Interval; IVW, Inverse variance weighted.
