# Supplementary material for: Genetically Supported Causality Between Micronutrients and Sleep Behaviors: A Two‐Sample Mendelian Randomization Study
Source: Brain Behav. 2025 Feb 5;15(2):e70237. doi: 10.1002/brb3.70237 (PMC11799067; doi:10.1002/brb3.70237)
Supplement: Supplementary file 4 — Supplementary Materials. [file BRB3-15-e70237-s002.docx]

Table S4. Two sample MR analysis of the association between circulating micronutrients and insomnia

| **Exposure** | **Method** | **No. of SNPs** | **P value** | ***OR* (95% CI)** |
| --- | --- | --- | --- | --- |
| Calcium | IVW | 20 | 0.14 | 0.98 (0.96, 1.01) |
|  | MR Egger | 20 | 0.81 | 0.99 (0.91, 1.08) |
|  | Simple mode | 20 | 0.77 | 0.99 (0.93, 1.06) |
|  | Weighted median | 20 | 0.42 | 0.99 (0.95, 1.02) |
|  | Weighted mode | 20 | 0.78 | 0.99 (0.92, 1.06) |
| Carotene | IVW | 15 | 0.52 | 0.99 (0.95, 1.02) |
|  | MR Egger | 15 | 0.43 | 1.03 (0.96, 1.11) |
|  | Simple mode | 15 | 1 | 1 (0.94, 1.06) |
|  | Weighted median | 15 | 0.85 | 1 (0.97, 1.04) |
|  | Weighted mode | 15 | 1 | 1 (0.94, 1.06) |
| Copper | IVW | 6 | 0.48 | 1 (1, 1.01) |
|  | MR Egger | 6 | 0.65 | 1 (0.98, 1.01) |
|  | Simple mode | 6 | 0.62 | 1 (0.99, 1.01) |
|  | Weighted median | 6 | 0.76 | 1 (0.99, 1.01) |
|  | Weighted mode | 6 | 0.99 | 1 (0.99, 1.01) |
| Folate | IVW | 13 | 0.47 | 0.99 (0.95, 1.02) |
|  | MR Egger | 13 | 0.91 | 1.01 (0.92, 1.1) |
|  | Simple mode | 13 | 0.52 | 1.04 (0.93, 1.15) |
|  | Weighted median | 13 | 0.9 | 1 (0.96, 1.05) |
|  | Weighted mode | 13 | 0.49 | 1.03 (0.94, 1.13) |
| Iron | IVW | 12 | 0.98 | 1 (0.96, 1.05) |
|  | MR Egger | 12 | 0.45 | 0.95 (0.82, 1.09) |
|  | Simple mode | 12 | 0.47 | 1.03 (0.96, 1.1) |
|  | Weighted median | 12 | 0.64 | 1.01 (0.97, 1.06) |
|  | Weighted mode | 12 | 0.47 | 1.03 (0.96, 1.1) |
| Magnesium | IVW | 17 | 0.46 | 0.99 (0.96, 1.02) |
|  | MR Egger | 17 | 0.45 | 0.98 (0.92, 1.04) |
|  | Simple mode | 17 | 0.37 | 0.97 (0.9, 1.04) |
|  | Weighted median | 17 | 0.59 | 0.99 (0.96, 1.03) |
|  | Weighted mode | 17 | 0.38 | 0.97 (0.9, 1.04) |
| Potassium | IVW | 14 | 0.65 | 0.99 (0.95, 1.03) |
|  | MR Egger | 14 | 0.54 | 1.04 (0.93, 1.16) |
|  | Simple mode | 14 | 0.52 | 1.02 (0.96, 1.1) |
|  | Weighted median | 14 | 0.48 | 1.02 (0.97, 1.06) |
|  | Weighted mode | 14 | 0.51 | 1.02 (0.96, 1.09) |
| Selenium | IVW | 6 | 0.12 | 1 (0.99, 1) |
|  | MR Egger | 6 | 0.71 | 1 (0.99, 1.02) |
|  | Simple mode | 6 | 0.48 | 1 (0.98, 1.01) |
|  | Weighted median | 6 | 0.46 | 1 (0.99, 1) |
|  | Weighted mode | 6 | 0.69 | 1 (0.99, 1.01) |
| Vitamin A | IVW | 12 | 0.1 | 0.62 (0.35, 1.09) |
|  | MR Egger | 12 | 0.5 | 0.46 (0.05, 4.03) |
|  | Simple mode | 12 | 0.88 | 1.1 (0.32, 3.85) |
|  | Weighted median | 12 | 0.63 | 0.82 (0.37, 1.82) |
|  | Weighted mode | 12 | 0.83 | 1.14 (0.35, 3.7) |
| Vitamin B12 | IVW | 9 | 0.83 | 1 (0.96, 1.03) |
|  | MR Egger | 9 | 0.6 | 0.97 (0.88, 1.07) |
|  | Simple mode | 9 | 0.88 | 0.99 (0.92, 1.07) |
|  | Weighted median | 9 | 0.59 | 0.99 (0.94, 1.04) |
|  | Weighted mode | 9 | 0.81 | 0.99 (0.92, 1.06) |
| Vitamin B6 | IVW | 17 | 0.2 | 1.02 (0.99, 1.06) |
|  | MR Egger | 17 | 0.15 | 0.95 (0.89, 1.02) |
|  | Simple mode | 17 | 0.49 | 1.03 (0.95, 1.1) |
|  | Weighted median | 17 | 0.45 | 1.01 (0.98, 1.05) |
|  | Weighted mode | 17 | 0.53 | 1.03 (0.95, 1.11) |
| Vitamin C | IVW | 10 | 0.15 | 0.97 (0.93, 1.01) |
|  | MR Egger | 10 | 0.65 | 1.03 (0.92, 1.14) |
|  | Simple mode | 10 | 0.27 | 0.95 (0.87, 1.04) |
|  | Weighted median | 10 | 0.17 | 0.97 (0.92, 1.01) |
|  | Weighted mode | 10 | 0.32 | 0.95 (0.87, 1.04) |
| Vitamin D | IVW | 13 | 0.24 | 0.98 (0.95, 1.01) |
|  | MR Egger | 13 | 0.79 | 0.99 (0.89, 1.09) |
|  | Simple mode | 13 | 0.54 | 0.98 (0.91, 1.05) |
|  | Weighted median | 13 | 0.3 | 0.98 (0.94, 1.02) |
|  | Weighted mode | 13 | 0.52 | 0.98 (0.92, 1.04) |
| Vitamin E | IVW | 12 | 0.45 | 0.98 (0.94, 1.03) |
|  | MR Egger | 12 | 0.5 | 0.96 (0.86, 1.07) |
|  | Simple mode | 12 | 0.47 | 0.97 (0.9, 1.05) |
|  | Weighted median | 12 | 0.24 | 0.97 (0.93, 1.02) |
|  | Weighted mode | 12 | 0.46 | 0.97 (0.91, 1.04) |
| Zinc | IVW | 8 | 0.34 | 1 (0.99, 1) |
|  | MR Egger | 8 | 0.87 | 1 (0.96, 1.03) |
|  | Simple mode | 8 | 0.94 | 1 (0.99, 1.01) |
|  | Weighted median | 8 | 0.51 | 1 (0.99, 1.01) |
|  | Weighted mode | 8 | 0.71 | 1 (0.99, 1.01) |

Abbreviation: No. of SNPs, number of single nucleotide polymorphisms; OR, odds ratio; CI: Confidence Interval; IVW, Inverse variance weighted.
