# Supplementary material for: Genetically Supported Causality Between Micronutrients and Sleep Behaviors: A Two‐Sample Mendelian Randomization Study
Source: Brain Behav. 2025 Feb 5;15(2):e70237. doi: 10.1002/brb3.70237 (PMC11799067; doi:10.1002/brb3.70237)
Supplement: Supplementary file 5 — Supplementary Materials. [file BRB3-15-e70237-s003.docx]

Table S5. Multivariable MR estimates of relationship between micronutrients and sleep chronotype

| **Exposure** | **Method** | ***Q*** | **difference** | ***P* value** |
| --- | --- | --- | --- | --- |
| Folate | MR Egger | 16.27795 | 11 | 0.131122 |
|  | IVW | 19.54196 | 12 | 0.076262 |
| Vitamin B6 | MR Egger | 17.68331 | 14 | 0.221587 |
|  | IVW | 18.6582 | 15 | 0.229643 |
| Vitamin D | MR Egger | 8.518787 | 10 | 0.578297 |
|  | IVW | 8.535948 | 11 | 0.664641 |
| Abbreviation: IVW, Inverse variance weighted | | | | |
